# Novel prevention insights into depletion of oxidative stress status through regular exercise and grape seed effective substance in heart ischemia rat model

**DOI:** 10.1002/fsn3.2714

**Published:** 2022-01-19

**Authors:** Safar Zarei, Farzaneh Taghian, Gholamreza Sharifi, Hassanali Abedi

**Affiliations:** ^1^ Department of Sports Physiology Faculty of sports sciences Isfahan (Khorasgan) Branch Islamic Azad University Isfahan Iran; ^2^ Research Center for Noncommunicable Diseases Faculty of Medicine Jahrom University of Medical Sciences Jahrom Iran

**Keywords:** antioxidants status, artificial intelligence tools, grape seed extract, myocardial ischemia, regular exercise

## Abstract

Myocardial ischemia (MI) is recognized as the most frequent cardiovascular disease which is the dominant cause of global morbidity and mortality. Artificial intelligence tools and integrative data analysis revealed superoxide dismutase, catalase, glutathione peroxidase, gap junction protein α, myosin heavy chains, and zinc finger transcription factor GATA4 are engaged in oxidative stress and in cardiomyopathy. Network analysis indicated that MAPK3 might be the highest distribution property and cut point in this network, which could be a potential candidate for preventing and treating oxidative stress in heart tissue. Among antioxidant agents, grape seed extract (GSE) is an effective substance that altered antioxidant status in heart tissue. Considering drug discovery methods, we illustrated that GSE might target the MAPK3 protein with sufficient binding affinity. Moreover, we found that low‐ and moderate‐intensity training might prevent the depletion of antioxidants after MI. GSE consumption altered the levels of superoxide dismutase, glutathione peroxidase, and catalase after 14 weeks. Therefore, the interaction of low‐ and moderate‐intensity training and GSE had a synergistic effect on the antioxidant status and relative expression of the *Mapk3*. Moreover, the interaction of high‐intensity training and GSE had a compensatory mechanism that could scavenge reactive oxygen species and improve endogenous antioxidants and modulate the *Mapk3* level in MI rats. Consequently, we displayed positive influence and synergic effects of simultaneous GSE prescription and regular physical activity for 14 weeks to prevent acute and chronic heart ischemia cardioprotective phenomenon. Furthermore, the capacitation oxidative stress and relative expression of the *Mapk3* was significantly increased by GSE and regular exercise.

## Graphical abstract

GSE consumption altered the levels of superoxide dismutase, glutathione peroxidase, and catalase. Physical activity and GSE had a synergistic effect on the antioxidant status and relative expression of the *Mapk3*. MAPK3 could be a potential candidate for preventing and treating oxidative stress in heart tissue.

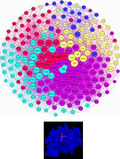

## 1 INTRODUCTION

Myocardial ischemia (MI) is considered the most frequent heart failure, which might be a severe challenge in modern medicine and the dominant cause of global mortality (Chen et al., 2021). Symptoms of MI are recognized as progressive thorax pain, nausea, vomiting, cardiac arrhythmia, feebleness, and fatigue. Based on the evidence, harmful environmental agents, including a sedentary lifestyle, smoking, and a high‐fat diet, enhanced the risk of MI (Xiang et al., 2021). Atherosclerosis due to the accumulation of plaques and artery wall injury in dyslipidemia is comprehended as critical events in the arterial thrombus (Hajibabaie et al., 2020). Disruption in cholesterol homeostasis with conservation of low‐density lipoproteins (LDL) led to activation of inflammatory cascades, which induced the recruitment of monocytes in inflammation sites (Andreou et al., 2015). This phenomenon directly increased heart cells’ reactive oxygen species (ROS) production. While the particular cardiomyopathy is not entirely understood, some molecular physiologists have suggested that chronic inflammation, apoptosis, and oxidative stress could play pivotal roles in MI pathogenesis (Xiang et al., 2021). Considering that ROS might be a notable pathological hallmark of many heart failure disorders, understanding the mechanisms through oxidative stress and mitochondrial dysfunction could identify novel therapeutic strategies (Panth et al., 2016). Significant efforts have been conducted to comprehend molecular mechanisms and are suggested effective therapeutics for the treatment and prevention of MI (Boström et al., 2010; Sy & Davis, 2010). Notably, several in silico studies have been evaluated to develop new therapeutic strategies for the disease (Ramírez et al., 2020).

Moreover, isoproterenol is a sympathomimetic beta‐adrenergic receptor agonist to induce the MI model. In addition, isoproterenol caused necrosis, myocardial infarction, mitochondrial dysfunction, and oxidative stress in the heart muscle (Hamilton et al., 2003). So far, no effective treatment strategy has been proposed to prevent MI. Some therapeutic approaches such as surgery and mesenchymal stem cells might adversely affect the function of the heart or require high technical equipment and costs (Naderi et al., 2019). Thus, management and prevention of MI might be a vital strategy to halt untoward effects of MI condition.

Physical activity and herbal drug consumption could be interesting preventive strategies to manage MI (Mittleman & Mostofsky, 2011). Based on the evidence, herbal drugs are considered a natural approach in managing MI, and prophylactic therapy with various bioactive nutrients and antioxidant‐rich compounds could positively influence antioxidant capacity, inflammation, apoptosis, and oxidative stress (Rubió et al., 2013). Studies have shown that proanthocyanidins or red grape seed extract (GSE) could have a protective antioxidant effect 20 and 50 times more than vitamin E and vitamin C, respectively (Brennan et al., 2000; Fernandes et al., 2015). Furthermore, recent studies have indicated that GSE could prevent cardiomyopathy and apoptosis by activating the natural antioxidant system in cardiomyocytes, reducing inflammatory factors levels, decreasing xanthine oxidase activity, and reducing oxidative stress (Belviranlı et al., 2012; Farías et al., 2017; Lian et al., 2016).

Exercise is recognized as the most effective nonpharmacological intervention for reducing cardiovascular disease (Ma et al., 2017). Growing evidence has shown that exercise might imply intensity and duration training, improving the ROS defense and decreasing lipid peroxidation leading to oxidant balance in the heart (Angadi et al., 2015; Waring et al., 2014). Furthermore, there are controversial results that regular exercise could not improve radical production during intensity training (Finkler et al., 2014; Rankovic et al., 2021). The mechanism responsible is related to the increased myocardial antioxidant capacity via increased antioxidant defense, reduced oxidative stress, and radical leak during oxidative phosphorylation (Du et al., 2007). In addition, evidence suggested that exercise could increase superoxide dismutase (SOD) and other antioxidant parameters and enhance the antioxidant defense (Du et al., 2007; Ntanasis‐Stathopoulos et al., 2013).

These aspects indicated that the intensity of exercise and antioxidant capacity might play a vital role in producing ROS and antioxidant status in the pathophysiology of MI. Therefore, evaluating the different intensity of exercise (low, moderate, and high) and interactive effect with GSE on the antioxidant status and prophylactic therapy in the rat‐induced MI by isoproterenol hydrochloride is required.

## 2 MATERIAL AND METHODS

### 2.1 Screening of genes related to heart ischemia

According to the list of the genes associated with heart failure (CUI: C0018801) in the DisGeNET database (Piñero et al., 2020), we have performed bioinformatics analysis to obtain hub genes involved in ischemia pathogenesis. We used the STRING 11.0 (Szklarczyk et al., 2021) database to construct the protein–protein network with the medium confidence threshold >0.4. Based on the visualized parameters of the network containing degree, betweenness, and closeness, 206 hub genes were collected among 1499 genes in the heart failure list. Classification of these hub genes based on the interaction and function was applied in Gephi software (Bastian et al., 2009), and identification of biological processes and molecular signaling pathways involved in the pathophysiology of ischemia was carried out based on enrichment (Xie et al., 2021) and literature mining. We engaged several databases such as the Kyoto Encyclopedia of Genes and Genomes (KEGG) (Kanehisa et al., 2017), Reactome (Jassal et al., 2020), panther (Mi et al., 2017), and STRING 11.0 (Szklarczyk et al., 2021) for the enrichment analysis to determine key pathogenicity pathways. KEGG pathway analysis considering *p* < .05 was enriched in KOBAS (Bu et al., 2021). Based on text mining data and identifying heart failure pathomechanism, we explored that antioxidant capacity in ischemia patients was impaired, which led to increased oxidative stress and inflammation and the development of myocardial infarction. These data suggested measuring antioxidant enzyme concentration and oxidative stress proteins after exercise and GSE consumption as a prevention protocol for MI. Moreover, the in silico analysis indicated that MAPK3 is a mediate protein between antioxidant enzymes and cellular compounds that influenced gene/protein expressions involved in heart pathogenicity.

### 2.2 Computational technique for molecular docking

Enrichment of hub proteins in several databases highlighted critical molecular signaling pathways involved in heart damage such as the TNF signaling pathway, MAPK signaling pathway, PI3K‐AKT signaling pathway, cell cycle, apoptosis, regulation of autophagy, oxidative stress, and inflammation (Dhingra et al., 2007; Ramos, 2008; Zhuang et al., 2011).

We found that based on the in silico analysis, MAPK3 had the highest betweenness centrality, and degree among hub proteins with significant differential expression. Moreover, MAPK3 could act as a regulatory factor in response to various signaling cascades such as inflammation, proliferation, differentiation, motility, survival, and metabolism (Rose et al., 2010). Also, the profibrotic effect of MAPK3 might induce the IL‐6 and TNF signaling pathways in cardiomyocytes. Hence, here, we engaged MAPK3 as a cut point protein in the PPI network involved in several signaling pathways and cellular processes associated with the pathogenesis of heart tissue. Based on this evidence, we proposed MAPK3 as a druggable target to drug design/discovery, pharmacophore modeling, and providing new therapeutic strategies.

We obtained the three‐dimensional structure of Mapk3 (ID 6GES in Protein Data Bank server) from the X‐ray diffraction method (Burley et al., 2021). Moreover, the SDF format of the GSE compound 3D structure was selected from the PubChem database (Kim et al., 2021). Optimization and preparation of macromolecule containing a deletion of extra chains, ligands, noncomplexed ions, solvent, and Gasteiger charges were performed in Chimera 1.8.1 software. Power and binding affinity between proanthocyanidin and Mapk3 protein were computed in search space with dimensions *x* = 64.7656, *y* = 54.4384, and *z* = 68.2176 Angstrom. In addition, a threshold of binding affinity <−5 and a root mean square deviation of atomic positions threshold <2 in PyRx software were considered (Dallakyan & Olson, 2015).

### 2.3 Animals and protocols

Fifty‐four male Wistar rats within the weight range of 160–180 g were provided and housed in the animal house of the Jahrom University of Medical Sciences. The rats were kept under a standard condition of 12‐h light/dark cycles, 22 ± 3°C temperature, and 50%–55% humidity. In addition, rats were fed standard chow and drank water ad libitum. This study complied with all the protocols of care and use of laboratory animals as approved by the Ethics Committee of the Jahrom University of Medical Sciences (ethical code: IR.JUMS.REC.1399.050). Rats were allowed to acclimate for 1 week before the initiation experiment. In the first step, before induced MI, rats exercised for 14 weeks (5 days/week) with different intensity (low, moderate, and high) and consumed 300 mg/kg GSE by gavage (five days a week; Jhun et al., 2013; Ni et al., 2012; Qin et al., 2020). In the second step, 24 h after the last interventions, the rats were induced the MI by isoproterenol hydrochloride (Iso‐hyd). Rats were randomly assigned into nine subgroups (*n* = 6): (1) myocardial ischemia rat as a positive control (MI), (2) rats with low‐intensity interval training (LIIT), (3) rats with moderate‐intensity interval training (MIIT), (4) rats with high‐intensity interval training (HIIT), (5) rats treated with grape seed extract (GSE), (6) rats treated with grape seed extract + low‐intensity interval training (GSE+LIIT), (7) rats treated with grape seed extract + moderate‐intensity interval training (GSE+MIIT), (8) rats treated with grape seed extract +high‐intensity interval training (GSE+HIIT), and (9) control without MI (Sed).

### 2.4 Exercise protocol

In this study, we designed three different protocol training, including low‐intensity interval training, moderate‐intensity interval training, and high‐intensity interval training based on the intensity and maximum oxygen uptake (Vo_2max_). For adaptation, the rats exercised running treadmills for 15 min at a 5‐m/min speed and a slope of 0 degrees in the first week. Then, all the rats exercised for 60 min a day, with different slopes and speeds to reach the different low, moderate, and high intensities. For each training session, 5 min were allocated to warm‐up and the last 5 min to cool down (Table 1). Overall, in the HIIT and MIIT programs, intensity, slope, and duration gradually increased in each session. In addition, speed (m/Mn) and VO_2max_, initiation from 10 (m/Mn), 52% VO_2max_ in MIIT, and 15 (m/Mn), 58% VO_2max_ in HIIT, respectively. The speed and VO_2max_ were progressively enhanced in each session. However, in the LIIT program, the slope was considered 0, and the speed and VO_2max_ began 7 (m/Mn), 40% VO_2max_, respectively (Ni et al., 2012).

**TABLE 1:** Exercise protocol

	Weeks													
	1	2	3	4	5	6	7	8	9	10	11	12	13	14
LIIT														
Speed	7	9	11	12	13	14	15	15.5	16	16.5	17	17.5	18	20
Slope	0	0	0	0	0	0	0	0	0	0	0	0	0	0
VO_2max_	45	55	57	57	57	57	59	59	59	59	59	60	60.5	61
MIIT														
Speed	10	15	17	19	21	23	25	27	29	30	30	30	30	30
Slope	1	2	3	4	4	4	4	5	5	5	5	5	5	5
VO_2max_	52	58	60	60	60	63	65	67	69	70	70	70	70	70
HIIT														
Speed	15	17	20	22	26	30	32	33	34	35	35	35	35	35
Slope	2	3	4	5	7	8	9	10	10	10	10	10	10	10
VO_2max_	58	60	65	66	67	73	79	79	87	90	90	90	90	90

### 2.5 GSE supplementation

300 mg/kg of GSE was dissolved in 1 cc of normal saline and administered by gavage for 5 days a week for 14 weeks (Jhun et al., 2013).

### 2.6 Induction of MI

Twenty‐four hours after the end of the 14 weeks of exercise and the oral administration of GSE, 80 mg/kg BW of isoproterenol hydrochloride was injected intraperitoneally into the rats (Frederico et al., 2009). The injection was repeated 24 h later. Four hours after the second injection, MI was induced. Subsequently, the rats were anesthetized with 50 mg/kg BW of ketamine hydrochloride and 10 mg/kg BW of xylazine hydrochloride. From the aorta and atrium, 4 ml of blood was collected. Serum was separated by centrifuging the collected blood samples at 1800 *g* (RCF) for 15 min. The serum were isolated and stored at −80°C until the biochemical parameters were measured. Moreover, we isolated the heart tissue of *Rattus* from all groups and stored it in cryotubes, speedily frozen, and preserved it at −80°C.

### 2.7 Serum measurement of oxidant and antioxidant

Serum levels of malondialdehyde (MDA, E0156Ra), total oxidant status (TOS, E1512Ra) as oxidant marker, and activity of superoxide dismutase (SOD, E1185Ra), glutathione peroxidase (GPx, E1759Ra), and catalase (CAT, E0869Ra) enzymes, as well as total antioxidant capacity (TAC) as an antioxidant marker was performed by commercially available enzyme‐linked immunosorbent assay (ELISA) kits (Bioassay Technology Laboratory) according to the manufacturer's recommendations.

### 2.8 RNA extraction and real‐time qPCR

To measure the gene expression levels of *Mapk3*, total RNA was extracted from the heart tissues of *Rattus* models by TRIZOL reagent based on manufacturer's protocol (Invitrogen). The NanoDrop spectrophotometer checked the quality and concentration of extracted RNA in 260/280 nm ratio (Thermo Fisher Scientific). The cDNA was synthesized by reverse transcriptase enzyme based on Fermentas kit protocol (Fermentas). The reverse transcription‐quantitative PCR (RT‐qPCR) was performed using the SYBR Green method following the manufacturer's protocol of the kit (Takara Bio, Inc.) by real‐time PCR cycler (Rotor‐Gene Qiagen). The primers are designed for *Mapk3* with sequence F: 5′‐CTGGAAGCCATGGAGAGATGTT‐3′, R: 5′‐TCGCAGGTGGTGTTGATAAG‐3′, and for internal reference gene B‐actin F: 5′‐CTTGCAGCTCCTCCGTCGCC‐3′ and R: 5′‐CTTGCTCTGGGCCTCGTCGC‐3′. Statistical analysis of relative gene expression of MAPK3 in comparison to b‐actin was calculated with the 2^−∆∆ct^ method.

### 2.9 Statistical analysis

Statistical analysis was calculated by GraphPad Prism Software (Version 9 Graph Pad Software Inc.). For normalizing distribution, the Kolmogorov–Smirnov test was used. Moreover, data were analyzed by two‐way and one‐way analyses of variance (ANOVAs) with Tukey's post hoc test due to multiple comparisons. Results were indicated as mean ± standard deviation (SD). Differences at *p* < .05 were considered to be significant in all analyses.

## 3 RESULTS

### 3.1 Biological features and in silico analysis

**FIGURE 1:**
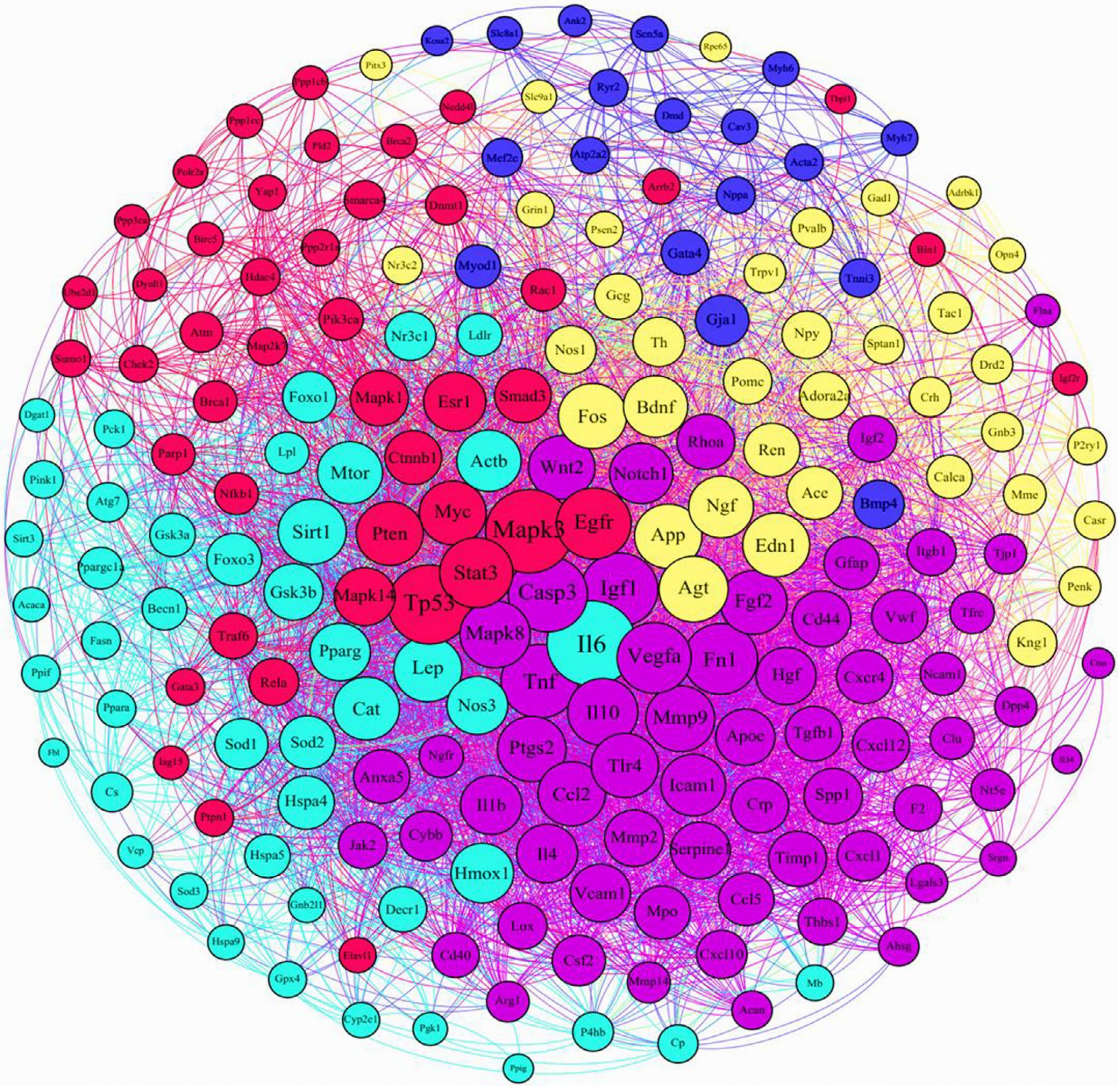
Hub genes classified in five clusters revealed that MAPK3 is a cut point between genes associated with oxidative stress/detoxification of oxidants and myocardial cell contraction

**FIGURE 2:**
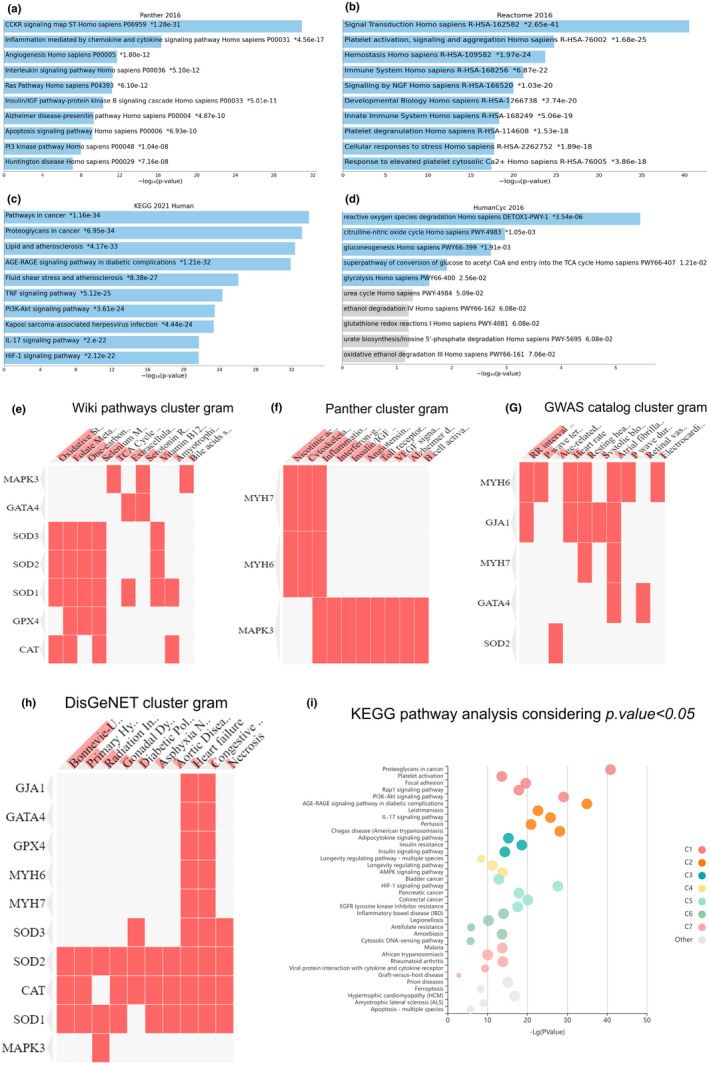
Enrichment of hub genes. (a–i) These genes are involved in several molecular signaling pathways such as PI3K‐Akt signaling pathway, AGE‐RAGE signaling pathway, lipid metabolism, TNF signaling pathway, inflammation mediated by chemokines and cytokines signaling pathway, interleukins signaling pathway, and insulin and insulin resistance signaling pathway, which led to alteration of antioxidant and oxidative stress capacity

Among 1499 genes existing in the heart failure list and based on the centrality parameters approach, we quantified 206 hub genes associated with cardiomyopathy in network analysis, and also these hub genes were classified in five clusters (Figure 1a). According to the biological network scanning, molecular associations related to key pathogenicity in heart tissue were characterized. This study sorted the genes involved in the oxidative stress pathway, such as detoxification of oxidants, arterial contraction, and fibrillation in vessel walls. Superoxide dismutase enzymes, catalase, glutathione peroxidase, gap junction protein alpha, myosin heavy chains, and zinc finger transcription factor GATA4 were located upstream of several genes in heart failure network (Figure 2a–h).

Furthermore, the in silico analysis demonstrated that Mapk3 had high centrality parameters in this network and was identified as a mediate protein concerning oxidative stress, antioxidant agents, and cellular contraction elements. Hence, we focused on the antioxidant capacity and stressed oxidative pathways involved in myocardial failure. Moreover, based on KEGG pathway analysis with *p* < .05 threshold, we showed that hub genes are involved in several molecular signaling pathways such as PI3K‐Akt signaling pathway, AGE‐RAGE signaling pathway, lipid metabolism, TNF signaling pathway, inflammation mediated by chemokines and cytokines signaling pathway, interleukins signaling pathway, and insulin and insulin resistance signaling pathway. Furthermore, literature surveys suggested that these pathways in heart failure pathogenesis remarkably altered antioxidant capacity and oxidants activity (Figure 2i).

### 3.2 The molecular docking feature survey results

**FIGURE 3:**
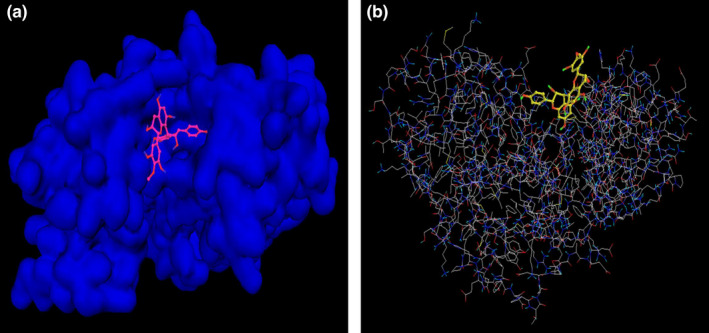
(a, b) Proanthocyanidins, an effective small molecule in grape seed, could target MAPK3 with sufficient binding affinity based on molecular docking methods

Proanthocyanidins, as a subgroup of flavonoids, are found in a wide range of plants (Tamba et al., 2007). Furthermore, antioxidant properties of proanthocyanins could reduce LDL oxidation, platelet aggregation arrest, and improve endothelial function (Al‐Rasheed et al., 2018; Lian et al., 2016; Tamba et al., 2007). Thus, we have selected GSE to evaluate the protective and preventive effects on MI. Furthermore, considering bioinformatics results, we predicted the power and binding affinity of proanthocyanidin in grape seed on the active site of MAPK3 protein as a target candidate to therapeutic and preventive approaches, which showed this small molecule with a strong and stable binding affinity (binding energy −7.9 kcal/mol and RMSD < 2 [upper and lower bonds]) interlock to Mapk3, and have functioned as a modulatory substance on the stress oxidative and inflammation pathways (Figure 3a,b).

### 3.3 GSE and exercise modulated the expression level of the *Mapk3*

**FIGURE 4:**
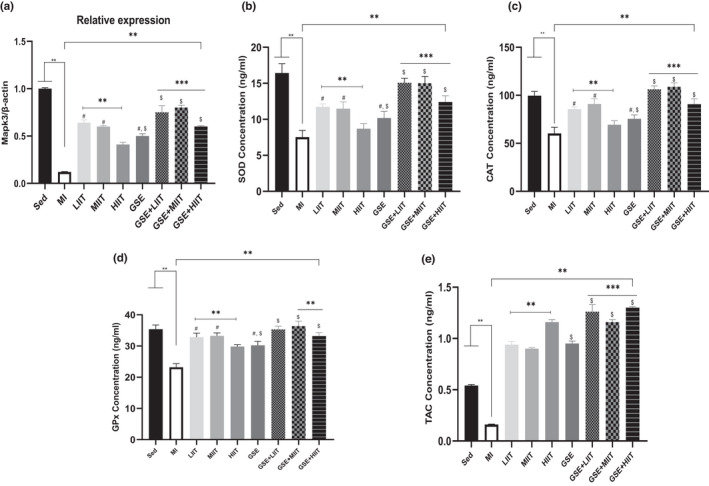
GSE and regular exercise halted the depletion of antioxidants and modulated the *Mapk3* after MI. (a) The relative expression of the *Mapk3* gene. (b–e) The concentration levels of superoxide dismutase (SOD), catalase (CAT), glutathione peroxidase (GPx), and total antioxidant capacity (TAC). *p < .05, **p < .01, ***p < .001. # represented HIIT groups, $ represented GSE groups

The relative expression of the *Mapk3* reduced in the MI rats (MI group) compared with the control rats (Sed group; Figure 4a). Moreover, the *Mapk3* level increased in rats with low‐intensity interval training (LIIT group), rats with moderate‐intensity interval training (MIIT group), and rats with high‐intensity interval training (HIIT group) compared to those of the MI group (Figure 4a). Notably, relative expression of the *Mapk3* was enhanced by low‐intensity interval training (LIIT group) and moderate‐intensity interval training (MIIT group) compared to the high‐intensity interval training group (HIIT group) (Figure 4a). Furthermore, the expression levels of the *Mapk3* was significantly high in rats treated with grape seed extract + low‐intensity interval training (GSE+LIIT group) and rats treated with grape seed extract + moderate‐intensity interval training (GSE+MIIT group) compared with other groups (Figure 4a). Based on these data, we revealed that the level *Mapk3* improved in rats treated with grape seed extract + high‐intensity interval training (GSE+HIIT group) compared with rats with high‐intensity interval training (HIIT group).

### 3.4 GSE and regular exercise prevented the depletion of antioxidants after MI

The concentration levels of SOD, CAT, and GPx predominantly decreased in MI compared with the control rats (Sed group; Figure 4b–d). Therefore, induction of MI altered the antioxidant status in MI rats. Although 14‐week regular exercise with a different protocol (low, moderate, and high intensities) offset the antioxidant level after inducing MI, the concentration level of SOD, CAT, and GPx significantly increased in rats with low‐intensity interval training (LIIT group) and rats with moderate‐intensity interval training (MIIT group) compared with high‐intensity interval training group (HIIT group; Figure 4b–d). Based on these data, we could conclude that low and moderate intensity training might prevent the depletion of antioxidants after MI. Furthermore, we found that the GSE consumption altered the SOD, CAT, and GPx levels after 14 weeks in rats treated with grape seed extract (GSE group; Figure 4b–d).

Interestingly, the concentration of SOD, CAT, and GPx are significantly elevated in rats treated with grape seed extract + low‐intensity interval training (GSE+LIIT group) and rats treated with grape seed extract + moderate‐intensity interval training (GSE+MIIT group) compared with other groups (Figure 4b–d). Therefore, the interaction of low and moderate intensity training and GSE synergistically affected the antioxidant status (Figure 4b–d). In addition, we found that the level of SOD, CAT, and GPx was enhanced in rats treated with grape seed extract + high‐intensity interval training (GSE+HIIT group) compared with rats with high‐intensity interval training (HIIT group) (Figure 4b–d). Hence, the interaction of GSE and high‐intensity training (GSE+HIIT group) had a compensatory mechanism that could scavenge ROS and improve endogenous antioxidants in MI rats (MI group).

Our data indicated that the total antioxidant capacity decreased in MI rats (MI group) compared to the control group (Figure 4e). In addition, we found that the TAC concentration was significantly enhanced in rats treated with grape seed extract (GSE group), rats with low‐intensity interval training (LIIT group), and rats with moderate‐intensity interval training (MIIT group) compared to those of the MI group (Figure 4e). On the other hand, the level of concentration of TAC was predominantly increased in rats with high‐intensity interval training (HIIT group; Figure 4e). Thus, high‐intensity interval training could elevate the antioxidant capacity after 14 weeks. Moreover, regular exercise combined with GSE consumption (GSE+LIIT group, GSE+MIIT group, and GSE+HIIT group) had a synergistic effect on the TAC concentration (Figure 4e). Hence, the level of TAC was enhanced by the interaction of GSE and regular exercise (GSE+LIIT group, GSE+MIIT group, and GSE+HIIT group), which prevented the excessive ROS accumulation in heart tissue.

### 3.5 Excessive ROS accumulation was declined by GSE and regular exercise

**FIGURE 5:**
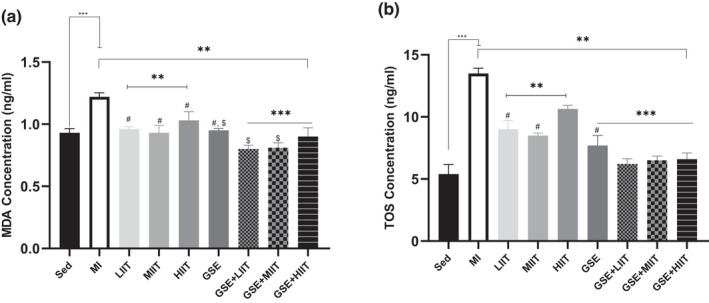
GSE and regular exercise decreased excessive ROS accumulation. (a, b) Serum levels of malondialdehyde (MDA) and total oxidant status (TOS). *p < .05, **p < .01, ***p < .001. # represented HIIT groups. $ represented GSE groups

In this study, the concentration of MDA was increased in MI rats compared with the control rats (Sed group; Figure 5a). In addition, 14‐week regular exercise (LIIT group, MIIT group, and HIIT group) declined the level of MDA compared with MI rats (MI group; Figure 5a). Notably, rats treated with grape seed extract (GSE group) significantly decreased the MDA concentration compared with MI groups (Figure 5a). Therefore, based on these data, GSE had scavenging properties that could diminish the free radicals of heart tissue. Moreover, rats treated with grape seed extract + low‐intensity interval training (GSE+LIIT group), rats treated with grape seed extract + moderate‐intensity interval training (GSE+MIIT group), and rats treated with grape seed extract + high‐intensity interval training (GSE+HIIT) significantly declined the excessive amounts of MDA, which indicated that these interactions had a synergistic effect (Figure 5a).

We found that the total oxidant status predominantly increased in the MI rats (MI group) compared with other groups (Figure 5b). Interestingly, after 14 weeks of regular exercise with different intensities (LIIT group, MIIT group, and HIIT group), the resistance to oxidant stress was significantly enhanced, leading to decreased ROS and oxidant status. Furthermore, TOS concentration in rats treated with grape seed extract + low‐intensity interval training (GSE+LIIT group), rats treated with grape seed extract + moderate‐intensity interval training (GSE+MIIT group), and rats treated with grape seed extract + high‐intensity interval training (GSE+HIIT) remarkably decreased compared with other groups, but GSE+HIIT was not significant compared with GSE+LIIT group and GSE+MIIT group (Figure 5b).

## 4 DISCUSSION

Atherosclerosis is a chronic immune‐inflammatory vascular disease and a significant cause of cardiovascular system dysfunction. Atherosclerotic lesions progress from fatty streaks accumulation to the complex, vulnerable plaques responsible for the acute outcomes of cardiomyopathy (Hajibabaie et al., 2020). The sediment of fatty streaks on vessel walls could affect by genetic factors, including familial hypercholesterolemia and harmful habits such as the high‐fat diet and sedentary lifestyle (Morisaki et al., 2009). The present integrative analysis was launched to assess the influence of grape seed prescription synchronous regular exercise training in different intensities (low, moderate, and high intensities) that could have a beneficial effect on preventing MI in rat models. According to the medical biology analysis and enrichment, hub genes were stratified in several molecular and cellular processes that pivotal molecular pathways involved in heart damage such as the TNF signaling pathway (Dhingra et al., 2007), MAPK signaling pathway, PI3K‐AKT signaling pathway (Zhuang et al., 2011), detoxification, cellular contraction in vessels (Ramos, 2008), cell cycle, apoptosis, regulation of autophagy, oxidative stress, and inflammation (Rose et al., 2010). These processes are associated with increased inflammatory factors (Neri et al., 2015), including activation of cytokines–chemokines and interleukins signaling pathways, AGE‐RAGE signaling pathway (Daffu et al., 2013), and apoptosis, which led to myocardial injuries. Growing evidence has revealed that oxidative stress and inflammation might be pivotal characteristics in metabolic disorders, heart failure, infarction, and reperfusion damage (Rahimi et al., 2021; Sverdlov et al., 2016; Yao et al., 2015).

Furthermore, oxidative stress is a key pathomechanism that led to progress in autophagia, apoptosis, and irreversible injury in cardiomyocytes (Xiang et al., 2021). Farías et al. (2017) reported that oxidative stress referred to the remarkable imbalance in normal oxidant scavenging enzyme systems leading to toxic intermediates cumulation and circulation. Based on immense evidence, the MAPK protein family is activated by ROS production, which mediated NF‐κB disjunction from its blocker and overexpress the NF‐κB pathway, subsequently triggering the apoptosis pathway and increasing the free radicals (Zhao, 2004). The bioinformatics result and text mining outcomes indicated that MAPK3 with the highest betweenness centrality and degree among hub proteins in the PPI network with significant differential expression could act as a regulatory factor in response to various signaling cascades such as inflammation, proliferation, differentiation, survival, and metabolism (Hernández et al., 2004). Also, the profibrotic effect of MAPK pathways might induce the IL‐6 and TNF signaling pathways and lead to reprogramming cardiomyocytes (Romero‐Becerra et al., 2020). Therefore, we suggested that MAPK3 as a druggable target to drug design/discovery could be a potential candidate for preventing and managing oxidative stress in heart tissue. MAPK3 is associated with oxidative stress/detoxification of oxidants and myocardial cell contraction in the protein–protein interaction network. On the other hand, MAPK3 is involved in several signaling pathways and cellular processes associated with the pathogenesis of heart tissue (Hao et al., 2020). Moreover, we showed that MAPK3 has the highest distribution property in this network which could affect the cardiomyopathy mechanism. In this study, we indicated that MI induction with isoproterenol hydrochloride significantly reduced the relative expression of the *Mapk3* gene. Previous evidence has demonstrated that the MAPK signaling pathway might play a pivotal role in MI (Huang et al., 2010). In addition, Hao et al. (2018) indicated that *Mapk3* gene MI was decreased.

In this study, consumption of the GSE altered the relative expression of the *Mapk3* gene. As a result, GSE significantly increased the level of a *Mapk3* gene. In addition, GSE prevented excessive ROS accumulation in heart tissue and might increase antioxidant capacity. Moreover, GSE had scavenging properties that reduced the free radicals (Aldubayan, 2020; Belviranlı et al., 2012). In addition, our data revealed that exercise training with different intensities (low, moderate, and high) increased the relative expression of *Mapk3*.

Interestingly, we found that the expression level of *Mapk3* in rats treated with grape seed extract + low‐intensity interval training (GSE+LIIT group) and rats treated with grape seed extract + moderate‐intensity interval training (GSE+MIIT group) significantly enhanced. Notably, the level of *Mapk3* was significantly amplified in rats treated with grape seed extract + high‐intensity interval training (GSE+HIIT group) compared with rats with high‐intensity interval training (HIIT group). Hence, the synergic effect of GSE interaction with three types of exercises (low, moderate, and high) remarkably enhanced a relative expression of the *Mapk3*. Based on the evidence, physical activity could mediate *Mapk3*, regulating inflammation and oxidative stress signaling pathways (Baghaiee et al., 2018; Belviranlı et al., 2012; Finkler et al., 2014).

Based on the text mining about herbal drug information, we found the influence of GSE on LDL oxidation, artery endothelial function, and platelet aggregation, which might remove free radicals by various mechanisms and result in less damage to the cardiovascular system during isoproterenol administration (Al‐Rasheed et al., 2018; Lian et al., 2016; Tamba et al., 2007). Furthermore, Aldubayan indicated that GSE consumption significantly decreased creatine phosphokinase, lactate dehydrogenase, creatine kinase as biochemical cardiac markers. Hence, cardiac injuries were reversed by GSE consumption (Aldubayan, 2020).

In this study, we found that GSE had scavenging properties that could remove the ROS of peroxide. We indicated that GSE significantly increased the SOD, CAT, GPx, and TAC concentrations. Moreover, the MDA and TOS concentration levels decreased by GSE consumption. Belviranlı et al. (2012) revealed that GSE significantly declined the concentration of oxidative markers, and the antioxidant status was predominantly raised compared with control and chronic and acute exercised groups.

Ample evidence has demonstrated that regular exercise has several beneficial effects, but it could have an adverse impact on the cells. Emerging evidence has shown that exercise might contribute to an imbalance between reactive oxygen, nitrogen species, and antioxidants, leading to excessive free radicals production during different types of exercise (Bloomer et al., 2005; Finkler et al., 2014). As a result, low‐, moderate‐, and high‐intensity exercises could alter the oxidative status. Our finding demonstrated that different exercise protocols (low, moderate, and high intensities) could offset the antioxidant level after inducing MI after 14 weeks of exercise training. In addition, these data indicated that the level of SOD, CAT, and GPx significantly enhanced in LIIT and MIIT groups compared with HIIT training, which prevented the depletion of antioxidants after MI.

Moreover, the concentration of oxidative markers (TOS and MDA) decreased compared with MI rats after the aforementioned protocol exercise. These data were in agreement with Rankovic et al. (2021) found that the HIIT and moderate exercise produced less ROS, and the level of oxidative stress markers was decreased. In addition, they indicated that the concentrations of superoxide anion radical and hydrogen peroxide in endurance training were enhanced, but these alterations were minimal in HIIT training. Notably, Jeremic et al. (2020) suggested that MAPK signaling pathways might be activated by exercise training and increase mitochondrial biogenesis and function. These data were in line with our artificial intelligence data, which showed that MAPK could be a pivotal modulator in oxidative stress and dynamic mitochondrial networks.

In this study, we demonstrated that interaction between GSE and physical activity had a synergistic effect on the relative expression of *Mapk3*, antioxidant, and oxidant status. Interestingly, we observed that the combination HIIT+GSE had a compensatory mechanism. In contrast to these results, Belviranl et al. (2012) indicated that the level of MDA and nitric oxide increased in the chronic and acute exercises along with GSE compared with control groups, and the antioxidant status was decreased.

Thus, according to artificial intelligence analysis and experimental evidence in this research, we displayed positive influence and synergic effects of simultaneous GSE prescription and regular physical activity for 14 weeks to prevent acute and chronic heart ischemia cardioprotective phenomenon. Besides, it could be inferred that low and moderate exercises followed by grape seed effective substance consumption as a compensatory mechanism in antioxidant defense. Interestingly, HIIT+GSE might increase the antioxidant capacity, improve the resistance to oxidant stress, and decrease the total oxidant status.

## CONFLICT OF INTEREST

None of the authors have any conflicts of interest to disclose.

## ETHICS APPROVAL AND CONSENT TO PARTICIPATE

This study complied with all the protocols of care and use of laboratory animals as approved by the Ethics Committee of the Jahrom University of Medical Sciences (ethical code: IR.JUMS.REC.1399.050).

## CONSENT FOR PUBLICATION

All authors support submission to this journal.

## Data Availability

All of the raw data and materials in the Islamic Azad University Isfahan (Khorasgan) branch are available upon request.
